# Current Advances of Three-Dimensional Bioprinting Application in Dentistry: A Scoping Review

**DOI:** 10.3390/ma15186398

**Published:** 2022-09-15

**Authors:** Nurulhuda Mohd, Masfueh Razali, Mariyam Jameelah Ghazali, Noor Hayaty Abu Kasim

**Affiliations:** 1 Department of Restorative Dentistry, Faculty of Dentistry, Universiti Kebangsaan Malaysia, Jalan Raja Muda Abdul Aziz, Kuala Lumpur 50300, Malaysia; 2 Department of Mechanical & Manufacturing Engineering, Faculty of Engineering & Built Environment, Universiti Kebangsaan Malaysia, Bangi 43600, Selangor, Malaysia; 3 DLima Dental Clinic, 44-A, Jalan Plumbum N7/N, Seksyen 7, Shah Alam 40000, Selangor, Malaysia

**Keywords:** 3D bioprinting, tissue engineering, cell-laden, bioink, dental tissue regeneration

## Abstract

Three-dimensional (3D) bioprinting technology has emerged as an ideal approach to address the challenges in regenerative dentistry by fabricating 3D tissue constructs with customized complex architecture. The dilemma with current dental treatments has led to the exploration of this technology in restoring and maintaining the function of teeth. This scoping review aims to explore 3D bioprinting technology together with the type of biomaterials and cells used for dental applications. Based on PRISMA-ScR guidelines, this systematic search was conducted by using the following databases: Ovid, PubMed, EBSCOhost and Web of Science. The inclusion criteria were (i) cell-laden 3D-bioprinted construct; (ii) intervention to regenerate dental tissue using bioink, which incorporates living cells or in combination with biomaterial; and (iii) 3D bioprinting for dental applications. A total of 31 studies were included in this review. The main 3D bioprinting technique was extrusion-based approach. Novel bioinks in use consist of different types of natural and synthetic polymers, decellularized extracellular matrix and spheroids with encapsulated mesenchymal stem cells, and have shown promising results for periodontal ligament, dentin, dental pulp and bone regeneration application. However, 3D bioprinting in dental applications, regrettably, is not yet close to being a clinical reality. Therefore, further research in fabricating ideal bioinks with implantation into larger animal models in the oral environment is very much needed for clinical translation.

## 1. Introduction

Defects in the craniofacial region including the alveolar bone can occur because of periodontitis, motor vehicle accidents, tumor and genetic factors. Periodontitis is the sixth most prevalent disease worldwide and the leading cause of missing teeth, followed by caries and trauma [1,2]. The dilemma of current clinical treatments in treating periodontitis cases is that therapies cannot repair the alveolar bone destruction and restore the functionality of the periodontally involved teeth [3]. In addition, the selection case of the suitable treatment such as guided tissue generation and bone graft strongly depend on the shape and size of the osseous defects. Moreover, rehabilitating the function of the oral cavity by means of dental implant in a severely resorbed alveolar bone may pose a challenge. Several approaches have been utilized for bone regeneration, such as employing the autogenous bone block, allograft and xenograft, however, these conventional treatments come with limitations. The drawbacks of these approaches include (i) donor site morbidity, lack of tissue availability, difficulty to shape and conform to the defect, and graft resorption of the autogenous bone [4,5,6]; and (ii) high rates of infection and increase risk of host immune response caused by allograft and xenograft [7]. These clinical challenges faced by clinicians and surgeons have led to the exploration of new technology in oral tissue engineering to fabricate functional dental tissue constructs, such as periodontal ligament, dentin–pulp complex and alveolar and craniomaxillofacial bone with patient-specific shape and size [8].

Three-dimensional (3D) bioprinting is an emerging combination technology of 3D printing and tissue engineering [9]. It is an ideal approach to fabricating customized complex 3D tissue constructs with defect-specific architectures through computer-aided design modeling to mimic native tissues [10]. It involves layer-by-layer precise deposition of cell-laden constructs from various biomaterials, cells and bioactive molecules with spatial control of the placement of functional components onto predefined locations (extracellular matrix, cells and pre-organized microvessels) [11,12,13]. The main advantage of 3D bioprinting is its ability to control the delivery of cells and materials in complex fabricated tissue-like structures. Hence, 3D bioprinted structures can provide cell-to-cell growth interconnectivity for better tissue regeneration [14].

**Figure 1:**
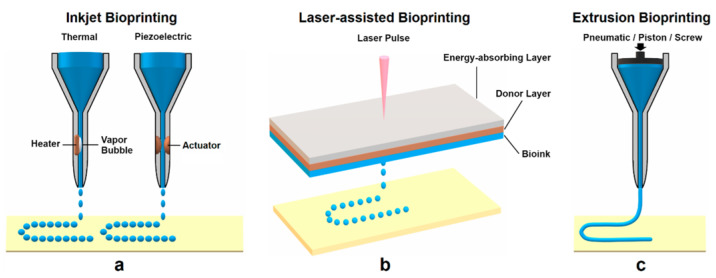
Common 3D bioprinting techniques: (**a**) inkjet bioprinting, (**b**) laser-assisted bioprinting (LAB) and (**c**) extrusion bioprinting [24].

The application of 3D bioprinting techniques that are widely used includes extrusion-based [15,16], inkjet-based [17], laser-assisted [18] and stereolithography [14], as shown in Figure 1. Extrusion-based bioprinting deposits the bioink either using a pneumatic, piston or screw-based system. It is the frequently preferred strategy for the development of multilayer scaffolds in tissue engineering because of the wide range of biomaterials selected for printing, such as natural and synthetic polymers, cell-laden hydrogel and cell aggregates [19,20]. In addition, it can manage high cell density, different material viscosities and crosslinking mechanisms [21]. Meanwhile, in inkjet bioprinting or drop-on-demand technique, it utilizes heating reservoirs, piezoelectric actuators, and electrostatic or electrohydrodynamic methods in order to deposit cells and/or biomaterials in the form of droplets onto the substrates. The advantages of this technique are fast printing speed and low cost. However, nozzle clogging caused by high cell density is one of the disadvantages of this method [11]. Laser-assisted bioprinting (LAB) utilizes a laser as the energy source and consists of an energy-absorbing layer, a donor ribbon and a receiving substrate [22]. This technology employs a noncontact bioprinting method and is nozzle-free, which can be used to deposit high viscosity bioink with a high resolution without nozzle clogging issues [11]. Although this approach results in high cell viability during printing, the effect of laser exposure onto the cells is still not known [23]. Stereolithography (SLA) uses ultraviolet light or an electron beam to initiate a polymerization reaction to place biomaterials onto a substrate. SLA is able to print complex architectures at extremely high resolutions. However, the drawbacks of SLA are its slow printing speed, high cost and limited selection of materials with suitable processing properties [24].

**Figure 2:**
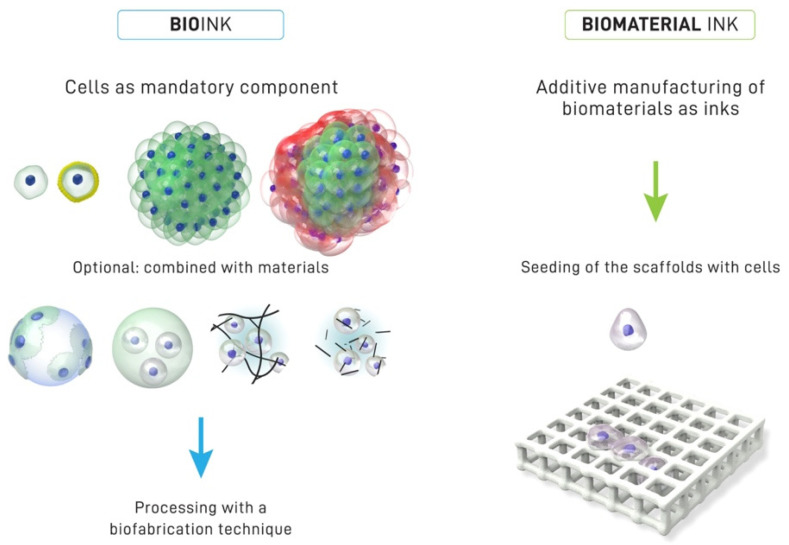
The characteristics distinction between bioink and biomaterial ink. In a bioink, cells are the mandatory component of the printing formulation, which can be in the form of single cells, coated cells and cell aggregates (one or several type of cells). The bioink may contain biomaterials and biologically active components. Meanwhile, the biomaterial ink is where the seeding cells are introduced within biomaterial scaffolds after printing. Reproduced with permission [25]. Copyright 2018 IOP publishing under a Creative Commons Attribution 3.0 Unported (CC BY 3.0). https://creativecommons.org/licenses/by/3.0/ (accessed on 21 August 2022).

One of the important components of 3D bioprinting is the bioink because of the effect it has on the outcome of the tissue engineering technology. Bioink refers to a formulation of cells that may contain biomaterials and biologically active components suitable for processing by an automated biofabrication technology [25] (see Figure 2). The use of bioinks enables the study of the effects of geometry and spatial organization on cell behavior and function in vitro, which can later be developed into in vivo models for applications in regenerative dentistry. At present, cell printing technology has become the preferred choice for a new biofabrication approach as compared to the conventional method of seeding cells on scaffolds. Three-dimensional bioprinting techniques are now able to incorporate living cells in bioprinted scaffolds, which enhance the position of cells. However, the disadvantage of the approach using scaffolds seeded with cells is that it could cause cell loss, which leads to poor cellular performance [26].

**Figure 3:**
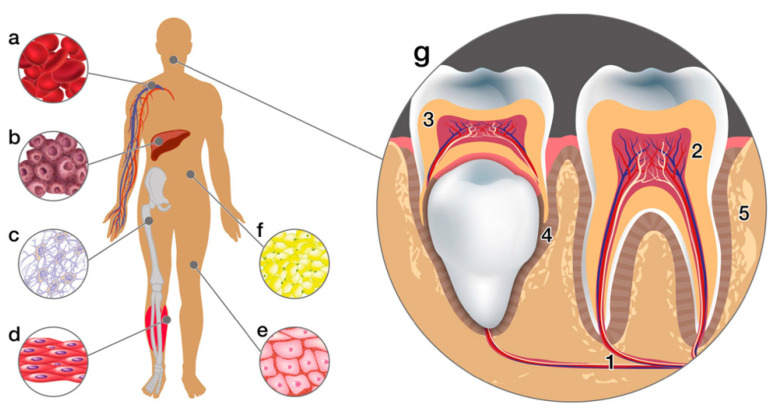
Sources of mesenchymal stem cells. This illustration shows human tissue sources: (**a**) peripheral blood, (**b**) liver, (**c**) bone marrow, (**d**) muscles, (**e**) skin, (**f**) adipose tissue and (**g**) dental tissues: (1. apical dental papilla, 2. dental pulp, 3. pulp from the exfoliated deciduous tooth, 4. periodontal ligament, 5. alveolar bone) [29].

Mesenchymal stem cells (MSCs), also known as “universal cells” are the most preferable cell source for tissue regeneration because they have self-renewal capability and can differentiate into various functional cell types under certain conditions [27,28]. MSCs can be isolated from embryonic stem cells or adult stem cells [29]. In addition, they are also easily extracted from almost all tissues (e.g., bone marrow, adipose tissue, umbilical cord and placenta), including dental tissues. Dental stem cells can be obtained from different parts of tissues such as periodontal ligaments (PDLSCs), dental pulp (DPSCs), from apical papilla (SCAPs) or exfoliated deciduous teeth (SHED) [28]. Rich sources of stem cells from the oral cavity have led to the great application and potential use in oral tissue engineering [28] (see Figure 3). Moreover, MSCs are also the most suitable cell source because of their immunomodulatory properties and ability to secrete protective biological factors [30,31].

The most common bioink materials are hydrogel-based bioprinted constructs. They have gained popularity in recent years because of similar characteristics to natural extracellular matrix (ECM), homogenous distribution of cells in the scaffolds, their ability to hold live cells, and enhancement of the cell viability in a hydrated 3D environment [32,33,34]. They can be derived from natural polymers (alginate, agarose, collagen, chitosan, gelatin, hyaluronic acid) or synthetic polymers including poly(ethylene glycol) (PEG), polyglycolic acid (PGA), poly(lactic-co-glycolic acid) (PDGA) and polycaprolactone (PCL). The advantages of natural polymers are the ability to biomimick ECM structure composition, the ability to self-assemble and also their biocompatibility [35], whereas, for synthetic polymers, they have proper degrading rate and photocrosslinking ability, which is not present in the natural polymer [36].

Three-dimensional bioprinting has emerged as a promising treatment strategy for fabricating complex biological constructs in oral tissue engineering, thus solving the issues associated with current therapies and overcoming the limitations of conventional techniques [37]. However, there is limited literature that has reported on the 3D bioprinting applications in dentistry. Therefore, this scoping review aimed to identify the gaps based on the available literature to answer the following questions: (i) How has 3D bioprinting technology been applied in dentistry? (ii) What are the types of biomaterials and cells used in 3D bioprinting?

## 2. Materials and Methods

### 2.1. Search Strategy

This review implemented the methodological framework from the Joanna Briggs Institute guidelines for scoping reviews and was carried out based on the Preferred Reporting Items for Systematic Reviews and Meta-Analyses extension for Scoping Review (PRISMA-ScR) [38,39]. The research questions for this review follow: (i) How has 3D bioprinting technology been applied in dentistry? (ii) What are the types of biomaterials and cells used in 3D bioprinting?

A search of the literature published through May 2022 was performed using four databases: Ovid, PubMed, EBSCOhost and Web of Science. The following search terms were used: (“3D bioprinting” OR “3D-bioprint*” OR “3D print*” OR “3D-print*” OR “Bioprinting” OR “Three-dimensional bioprint*”) AND (“Tissue engineering” OR “Tissue regeneration” OR “Bone regeneration” OR “Regenerative medicine” OR “Periodontal regeneration” OR “Guided tissue regeneration”) AND (“Dental” OR “Dentistry”). Additional records were identified through a manual search of the references lists. The search was limited to articles in the English language and had no restriction on the time frame of publication year.

### 2.2. Study Selection

The initial screening of the identified studies was conducted based on the information in the titles and abstracts by two independent reviewers (N.M. and M.R.). In addition, the full text of potentially eligible studies was retrieved for further screening of their suitability determined by inclusion and exclusion criteria. Any disagreement between reviewers on study selection was resolved by a third reviewer (N.H.A.K.) through discussion.

The inclusion criteria for the included studies were defined based on the Participant/Population (P): cell-laden 3D-bioprinted construct; Concept (C): intervention to regenerate dental tissue using bioink that incorporates living cells or also in combination with biomaterial and/or growth factors before or during printing; Context (C): application of 3D bioprinting tissue-engineered in the dental field. However, studies were excluded if they were case reports, review papers or conference abstracts. Articles that reported cell seeding of the scaffolds after printing and were not related to the dental application were also excluded.

### 2.3. Data Extraction and Analysis

Extraction and synthesis of information from the included studies were summarized and presented into a table of evidence by the first reviewer (N.M.) and verified by the second reviewer (M.R.) to ensure that they were aligned with the research questions. The extracted data of the included studies were publication details (first author, year of publication and country of study), study design (in vitro and in vivo), 3D bioprinting strategy (type of 3D bioprinter and parameters of 3D printing technique), materials, type of cells, animal models characteristics (animal species, gender, age, weight and defect size), and application in dental field and outcomes of the 3D bioprinting.

## 3. Results

### 3.1. Study Selection and Characteristics

**Figure 4:**
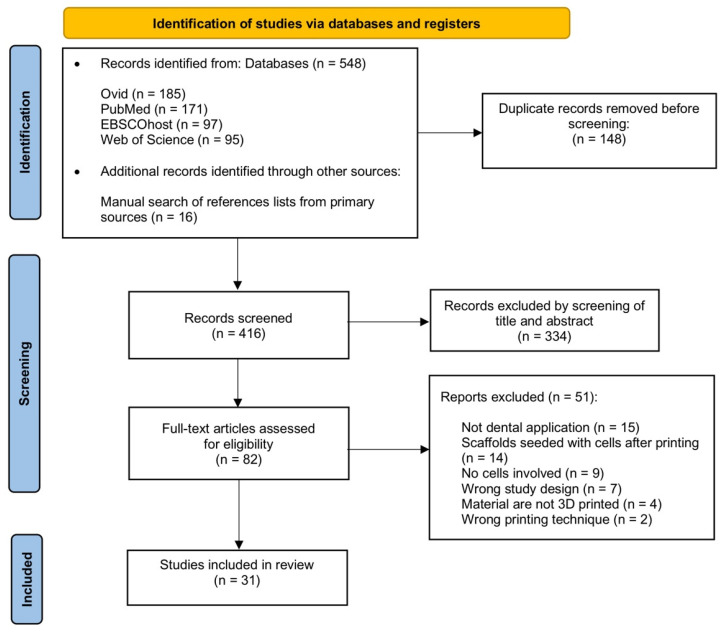
PRISMA flow diagram depicting the results of the search strategy.

This revised search strategy generated 548 records from four databases: Ovid (*n* = 185), PubMed (*n* = 171), EBSCOhost (*n* = 97) and Web of Science (*n* = 95) through May 2022. In addition to electronic databases, a manual search of reference lists was carried out through primary sources and additional eligible studies were added (*n* = 16). Out of these, a total of 148 duplicates were excluded and 334 records were assessed based on their titles and abstracts. This was performed by using the online literature review application, Rayyan software (http://rayyan.qcri.org (accessed on 9 September 2022)) [40]. Moreover, full texts of the 82 articles were retrieved for eligibility based on the inclusion and exclusion criteria. Out of those, 51 were further excluded because the articles were not for dental application (*n* = 15), scaffolds seeded with cells after printing (*n* = 14), no cells involved (*n* = 9), wrong study design (*n* = 7), materials are not 3D printed (*n* = 4) and wrong printing technique (*n* = 2). Finally, there were 31 articles included in this review, as recorded in Figure 4.

### 3.2. Characteristics of Included Studies

A third of the included articles were conducted in the USA (*n* = 10) [41,42,43,44,45,46,47,48,49,50]. It was followed by Korea (*n* = 5) [51,52,53,54,55], France (*n* = 4) [56,57,58,59], Germany (*n* = 3) [60,61,62], China (*n* = 3) [63,64,65], Taiwan (*n* = 2) [66,67], Canada (*n* = 1) [68], Australia (*n* = 1) [69], Sweden (*n* = 1) [70] and Japan (*n* = 1) [71]. The frequency of publications showed a steady rise from 2015 to the present time, thereby reflecting a growing interest in the 3D bioprinting technology in the dental field. The main characteristics of the included studies are described in Table 1.

### 3.3. Three-Dimensional Bioprinting Strategy for Dental Application

**Figure 5:**
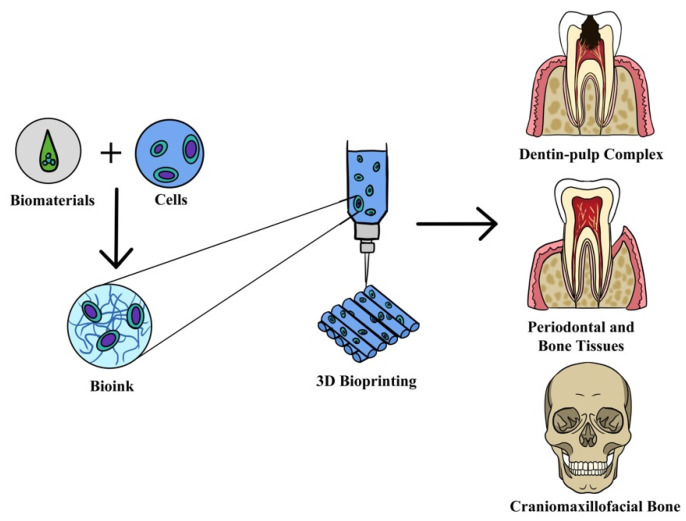
Three-dimensional bioprinting strategy for dental application such as regeneration of dentin–pulp complex, periodontal, alveolar bone tissues and craniomaxillofacial bone.

Nearly two-thirds of the research reported in this review used extrusion-based 3D bioprinting technique to fabricate scaffolds. This technique was used in eight studies for bone regeneration application [41,42,46,48,49,50,65,70], four studies used for general dental tissue regeneration [47,52,54,55], another three for periodontal ligament [53,66,69] and followed by dentin and dental pulp regeneration [43,51,67]. Apart from regeneration application, extrusion-based technique has also been used to explore the usage of scaffolds for head and neck cancer in vitro models [68]. For laser-assisted bioprinting, all the studies utilized this technology for bone regeneration [56,57,58,59]. However, for inkjet-based technique, there was various usage for regeneration of periodontal ligament [63], dental pulp [60] and bone [64]. Meanwhile, the other technique, stereolithography, has been used for bone regeneration [62] and alveolar bone in vitro modeling [61]. Another 3D bioprinting technique, which is a scaffold-free method, 3D tissue spheroids (cell aggregates) bioinks were developed by skewering individual cellular spheroids into a predetermined design onto a needle-array platform without any supporting hydrogel or matrix. This technique has been employed for periodontal ligament [71] and bone regeneration [44,45]. Overall, half of the studies used 3D bioprinting for alveolar bone/bone regeneration for dental tissue engineering application. Figure 5 shows 3D bioprinting in dental applications. The other information, such as the type of bioprinters and 3D bioprinting, is presented in Table 2.

### 3.4. Bioinks for 3D Bioprinting

In this review, the majority of cell-laden bioinks consist of combinations of two to four polymers and/or biomaterials for 3D bioprinting applications. The commonly used materials for the fabrication of bioinks were natural polymers (collagen, gelatin, fibrin, alginate, hyaluronic acid (HA), chitosan, agarose and glycerol). Naturally derived polymers with chemical modifications such as gelatin methacryloyl (GelMA) and methacrylated hyaluronic acid (MeHA) also have been used as bioinks. Only one study used synthetic polymer alone, Poloxamer-407, a synthetic copolymer of poly(ethylene glycol) and poly(propylene glycol) [54]. Meanwhile, three studies used hybrid materials that are the combination of GelMA and poly(ethylene glycol) dimethacrylate (PEGDA) [61,63,64].

Decellularized extracellular matrix (dECM)-based, also termed tissue-specific bioink, was used by two studies [52,55]. In addition, some studies added bioceramics materials such as nano-hydroxyapatite [49,50,56,65], calcium phosphate [55] and calcium silicate [59,67] with composite bioinks. Bone morphogenetic protein (BMP) was the most commonly used growth factor reported in this review [47,49]. Other growth factors such as vascular endothelial growth factor (VEGF) [58] and fibroblast growth factors (FGF) [53] have also been investigated within 3D bioprinted constructs. Meanwhile, one study utilized gene-based growth factors using a nonviral gene delivery method, which was the combination of platelet-derived growth factor-B encoded plasmid DNA (pPDGF-B) and bone morphogenetic protein-2 encoded plasmid DNA (pBMP2) [50].

In 3D bioprinting, the crosslinking approach is an important aspect to achieve the biomechanical stability of 3D constructs. Herein, the collagen-based bioinks were crosslinked either using temperature [53,60] or physical [66], or a combination of both [49,50], or genipin [55]. Eight studies used GelMA, the modified naturally derived polymer, which was crosslinked by photopolymerization [46,47,54,61,62,63,64,67,69]. Synthetic polymer, Poloxamer-407 also uses UV light for photocrosslinking [54]. Apart from that, alginate bioink used calcium chloride as its crosslinker [43,65,68,70]. Fibrin-based bioink can be made from fibrinogen by enzymatic reaction of thrombin [41,51,52].

### 3.5. Cells for 3D Bioprinting

Types of cells for 3D bioprinting reported in this review were mesenchymal stem cells and cell lines. Stems cells isolated from the human oral cavity have been used, such as periodontal ligament stem cells (PDLSCs) [53,63,65,69], dental pulp stem cells (DPSCs) [47,48,51,52,55,60,67] and stem cells from apical papilla (SCAPs) [43,54,57,58,59]. Meanwhile, one study used gingival fibroblast in the cell-laden bioink [66]. In this review, human dental stem cells were isolated from third molar teeth of young healthy patients with an age range of 18–28 years old. Only one study isolated nonhuman periodontal ligament stem cells from rats [64].

As reported in this review, other main sources of cells used were nondental-origin stem cells from bone marrow [44,45,46,49,50,62] and adipose tissue [42,70]. Apart from this, some studies used extracted cells derived from bone [61,62], periosteum [62], amniotic fluid [41] and umbilical vein [58,60,61]. These MSCs sources were from humans and various animals such as rats, mice and porcine. Furthermore, two studies implemented a co-culture approach using SCAPs and human umbilical vein endothelial cells (HUVECs) [58], DSPCs and HUVECS [60] in their research.

Other types of cells that have been used were human squamous cell carcinoma lines from cancer larynx (UM-SCC-12) and tonsillar pillar (UM-SCC-38) [68], multipotent clonal human PDL cell line (line 1–17) [71] and mouse bone marrow stromal precursor D1 cell line [56]. Herein, 3D bioprinting produces high cell viability after printing in the range of 70% to greater than 95%. The details of the type of cells used in 3D bioprinting are presented in Table 3.

### 3.6. In Vivo Application in Dental Tissue Engineering

Out of 31 studies, a total of 11 studies reported in vivo applications on animal models. However, only nine studies used cell-based scaffolds and the other three were cell-free bioprinted constructs implanted in vivo using the extrusion-based technique. Therefore, in this review, only nine studies were reported for in vivo evaluation, which involve implantation of the 3D bioprinted constructs into calvarium [41,53,56,57,58,59,66], alveolar bone [64] and subcutaneous area [55]. The calvarial bone defects were surgically created without penetration into the dura with a diameter ranging from 3.3 to 8 mm. In addition, the alveolar defect was created with a dimension of 4 mm length × 3 mm width × 2 mm height. One study reported implantation of bioprinted constructs (8 × 8 × 4 mm^3^) on dorsal subcutaneous pockets. Meanwhile, for animal models in this review, only one article used rabbits as osteoporotic models in their study [66], whereas the others used immunodeficient rats or mice (either athymic, balb/c, NOG or NSG mice) as their animal models [41,53,55,57,58,59,64].

**Figure 6:**
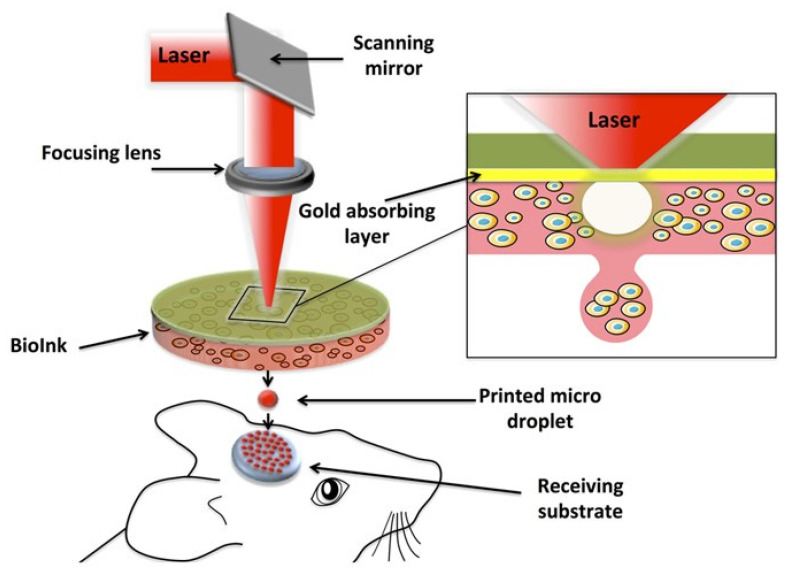
Intra-operative bioprinting (IOB) using laser-assisted bioprinting (LAB) approach in vivo application. LAB setup comprises a pulsed laser beam, a ribbon (transparent glass slide coated with a laser-absorbing layer of metal) and a receiving substrate. Reproduced with permission [56]. Copyright 2017 SpringerNature publishing under a Creative Commons Attribution 4.0 International (CC BY 4.0). (https://creativecommons.org/licenses/by/4.0/ (accessed on 21 August 2022)).

Moreover, four studies reported performing in situ or intra-operative bioprinting of the 3D constructs during surgical intervention on the cranial bony defects using laser-assisted bioprinting, as shown in Figure 6 [56,57,58,59]. After implantation of the 3D printed constructs, the animals were euthanized at time points ranging from 3 to 20 weeks to harvest implanted specimens. The characteristics of the animal models are summarized in Table 4.

## 4. Discussion

Three-dimensional bioprinting has become an advanced tissue engineering approach to create dental tissue constructs to address the need for regenerative dentistry. The studies included in this review showed a wide range of heterogeneity in terms of different types of novel bioinks, 3D bioprinting techniques, type of cells used and applications of 3D bioprinting in dentistry.

In addition, recent 3D bioprinting development provides multiple approaches for the biofabrication of tissue constructs within scaffolds or scaffold-free environments. This approach could produce 3D structures with spatial organization of cells that facilitates the control of the shape of regenerated tissues. However, 3D bioprinting still faces significant challenges as compared to the nonbiological printing approach in terms of more complex architectural fabrication and the stability of cell behavior. In this review, the extrusion-based technique is the most common 3D bioprinting method for dental application. This technique is widely used because it is cost-effective and able to replicate complex tissue structures using a wide variety of biomaterials and cell types [19,20,72]. Moreover, the extrusion-based techniques can produce cell-laden bioinks in the form of continuous strands or fibers, which enable fabricating of large-scale 3D scaffold constructs [15,73]. Furthermore, printing parameters such as printing speed, pressure, resolution, temperature, nozzle inner diameter, scaffold design and viscosity of the bioink are important factors in determining the uniformity of continuous strands deposition of the bioprinted scaffolds [74].

Bioink is also an important component of 3D bioprinting. The ideal bioink formulation should satisfy certain biomaterial and biological requirements. Biomaterial properties include printing compatibility, mechanical properties, biodegradation, modifiable functional groups on the surface and post-printing maturation, whereas the biological requirements mainly include biocompatibility, cytocompatibility, and bioactivity of cells after printing to support and maintain cellular viability and function [36]. Therefore, the treatment outcome of the tissue regeneration depends on the bioinks used. Nonetheless, at present there is a lack of ideal 3D printable bioinks focused on dental tissue regeneration.

Natural polymers are the most common type of polymer used as bioink because they have a similar native composition as the ECM, biocompatibility and biodegradation properties, together with established interactions between natural polymers and cells [75]. Collagen type I is a hydrogel of choice for tissue engineering, which agrees with the research reported in this review. In addition, it is the most abundant component of the native ECM and provides an encouraging environment for cell adhesion and proliferation [76]. Crosslinking collagen matrices play an important role in the strength and stability of the structure. In comparison to noncrosslinked collagen, there is an increase in tensile strength and viscoelastic properties when using a crosslinker [77,78]. The crosslinked collagen constructs demonstrated different stiffness strengths based on types of oral tissue engineering. However, for dental pulp tissue application, the combination of collagen and agarose showed a storage modulus of approximately 0.03–0.3 kPa [60]. A study by Moncal et al. showed that in calvarial bone repair, the storage modulus of the collagen-based bioink was 8.2 ± 1.4 kPa [49]. In another study for dental tissue engineering application, collagen/β-TCP 20 wt% showed 27.9 ± 2.2 kPa modulus, which was higher than collagen alone because of the added bioceramics in the bioink [55]. The balance between mechanical strength and cell viability of the 3D constructs is crucial to maintaining cell structure and promoting cell growth. The natural polymer can be combined either with synthetic or another type of natural polymer to produce a more stable construct with enhanced function and properties. Another hydrogel-based bioink that shows potential in 3D bioprinting is GelMA because of its superior biocompatibility and photocrosslinking properties [79]. Herein, various GelMA-based bioinks have been developed to fabricate tissue structures for application in periodontal ligament [63,69], dentin [67], bone [42,46,62,64] and dental tissue regeneration [47], along with in vitro modeling of alveolar bone [61].

Synthetic polymers can be manufactured in large quantities and have longer shelf life as compared to natural polymers [80]. The photocrosslinking ability and controllability of mechanical properties, degradation rate, pH and temperature are among the advantages of using the polymers. However, most synthetic polymers lack the ability to promote cellular adhesion and recognition, and have limited biodegradability and biocompatibility, which restrict their usage in clinical applications [81]. Poly(ethylene glycol) (PEG) is one of the most popular synthetic polymers in tissue engineering [82]. PEG-based bioink can be modified using diacrylate (DA) or methcrylate (MA) groups to improve mechanical strength. In addition, the combination of PEGDA/GelMA has been used for periodontal ligament and bone regeneration application [63,64] and for in vitro alveolar bone models [61]. Moreover, a combination of natural and synthetic polymers can be a promising bioink material for fabricating biomimetic tissues because of their combined properties [83]. Another bioink, dECM, has been frequently used as a bioink in 3D bioprinting because of its good inductive property that can promote cell proliferation and differentiation together with the interaction between cells to cells and cells to ECM [84,85]. Herein, the various types of novel bioinks demonstrated high printability and cell viability, which have the potential in dental tissue regeneration applications. However, a few studies showed that novel bioinks need formulation adjustment for oral tissue engineering: (i) collagen-based with TCP (BioRoot RCS^®^, Septodont, France) bioink did not demonstrate regenerative potential in a calvaria critical bone defect model [59], (ii) combination of collagen-based with β-TCP reduced the capability of osteogenic differentiation, mineralization and vascularization compared to dECMs with β-TCP [55] and (iii) addition of FGF-2 to the collagen bioink did not play a role in periodontal ligament regeneration [53].

The use of growth factors in 3D bioprinting is not prevalent in dental applications because of the additional complexities that may arise. In general, the strategies in utilizing the growth factor in tissue engineering are still unclear mainly because of the uncertainties of the delivered dosage in vivo by the constructs [86], the effects of multiple uses of growth factors [87], and no standardization and arbitrariness of growth factor dosage from the broad range of concentrations available [88].

Three-dimensional bioprinting technology with the support of stem-cell-containing scaffolds has emerged as an alternative treatment strategy to address the critical need for dental tissue regeneration [37]. This is because 3D bioprinting of the cell-laden hydrogel combines physical and biological properties to attain a 3D composite construct with homogenous cell distribution, proliferation and differentiation [89]. Adult stem cells are currently the most common cells used in the field of bone tissue engineering. The advantage of stem cells derived from dental tissues is that they are easily accessible and have interesting proliferation and differentiation abilities. Healthy tissues and young patients contain a large number of normal stem cells as compared to inflamed or traumatized tissues and aging patients, which can affect the potential for tissue repair [90].

In addition, dental pulp is highly vascularized; thus, it poses a major challenge in regenerating dental pulp tissues. DPSCs are a promising source for odontogenesis because of their excellent clonogenic efficiency [91] and proangiogenic capacity [92]. A study by Duarte Campos et al. has shown evidence of successful vascular tube formation using printable bioink that contains co-cultures of human umbilical vein endothelial cells (HUVEC) with DPSCs [60]. These co-cultures not only can enhance angiogenesis but also stabilize the capillary-like structures [93]. Another study also showed promising results with DPSCs, demonstrating spatial regulation of odontogenic differentiation for 3D dentin–pulp complex formation [51]. Apart from DPSCs, SCAPs isolated from immature apical papilla could enhance odontogenic differentiation, which in the future could engineer dentin–pulp tissues [43].

Periodontium is a complex structure consisting of the periodontal ligament, cementum, gingiva and alveolar bone. Designing a scaffold for periodontal regeneration would require multilayer cementum–periodontal ligament–alveolar bone components to achieve both hard and soft tissue regeneration. The biomaterials should have a combination of polymers (i.e., collagen and gelatin) and inorganic components (i.e., hydroxyapatite, calcium phosphates and bioactive glass), given that they have different mechanical strengths [94]. However, only one study in this review used a bilayered scaffold, which consisted of collagen and strontium-doped calcium silicate for periodontal regeneration [66]. Meanwhile, the others used GelMA-based PDLSCs as their bioinks for periodontal ligament regeneration application [53,63,69]. Furthermore, PDLSCs can facilitate the formation of new alveolar bone and functional ligaments in damaged periodontal tissue under proper stimulation [95,96,97].

In craniomaxillofacial reconstruction, the patient-specific shape is the key factor for clinical application as there are no similar defects in terms of size and shape. Hence, achieving facial symmetry is a crucial outcome to prevent problems such as aesthetics, articulation and mastication. Thus, 3D bioprinting is favorable in fabricating specific dimensions of 3D constructs with targeted regeneration of complex tissue architectures to address the reconstructive challenges [98]. Meanwhile, in dental applications for bone regeneration, stem cells from dental origin are popular cell sources in this review. DPSCs have shown to have higher osteogenic potential than bone marrow stem cells (BMSCs), and can also produce vessel-integrated bone tissue structures which are imperative for large bone defect reconstruction [48]. The third molar is the best source for DPSCs and it can proliferate and differentiate into osteoblast and odontoblast lineages to form dentin and bone [99,100]. Other cell types that have been used are PDLSCs, which have shown multidirectional differentiation to form alveolar bone and cementum for bone tissue regeneration [101].

For the research reported in this review, bone marrow stem cells that have been used were mostly sourced from rats and mice. If human-sourced bone marrow were to be used for clinical translation for oral and craniofacial defect regeneration, it presents a few disadvantages, such as painful harvesting of bone marrow procedure and the issue of harvest yield [102]. Hence, human adipose tissue presents a desirable choice for tissue regeneration considering the simple harvesting process as compared to the traditional method. It also causes less morbidity in the patient and provides an abundant amount of adipose stem cells [103,104]. Another advantage is that the cells are capable to differentiate into osteoblastic lineage [103].

Furthermore, a stable printed scaffold with viable cells which can withstand the load-bearing force is one of the contributing factors to the predictable outcome of reconstructing oral and craniofacial defects. Therefore, in the research reported in this review, the crosslinking mechanism has been used to increase the stability of materials such as photocrosslinking of GelMA bioinks [42,46,62,64]. Another strategy is by combining bioceramic materials such as nano-hydroxyapatite, calcium phosphate and calcium silicate to gain improved mechanical properties of the constructs [105]. Given that hydroxyapatite exhibits the same function and composition as bones and teeth [106], the addition of hydroxyapatite or tricalcium phosphate to form 3D osteogenic structures has been widely explored in this field because the materials mimic the inorganic component of bone tissue [76,106].

In addition, scaffold-free tissue engineering is another 3D bioprinting technology to fabricate tissue construction. As reported in this review, this approach has been utilized for periodontal ligament [71] and bone regeneration application [44,45]. This technique does not use exogenous scaffolds for support but relies on generating constructs from cell spheroids fusion because of the cell-to-cell contact behavior [107]. Moreover, it eliminates the degradation time factor of scaffold materials, which can affect the viability of the encapsulated cells caused by byproducts of fast degradation scaffolds, whereas the slow degradation time may hinder the matrix formation [108,109]. Hence, using the scaffold-free method, cells would secrete the extracellular matrix required to provide structure. Therefore, the cells are within a biologically optimized extracellular matrix (ECM) environment to which they are suited. The utilization of cell-secreted ECM also eliminates the need to rely on the degradation of synthetic scaffold materials [45].

Meanwhile, for in vivo utilization, the studies used immunodeficient rats or mice as their animal models because these models are excellent recipients for the engraftment of human cells [58]. Small animal models are a popular selection for in vivo studies because of their ease of handling and lower cost to manage [110]. The prominent dissimilarity to the human bone [111] and the healing after implantation in small tissue defects in small animals [9] indicates that the results should be interpreted with caution, and thus, it plays a small role in translating the findings into human clinical applications [112,113,114]. The critical-sized calvarial bone defect has been widely used to study the interaction between cells and biomaterial on bone regeneration [115]. In addition, in situ bioprinting or intra-operative bioprinting is an advanced technology that has been performed to repair the defect via the bioprinting process on a live subject during the surgical intervention [15,116]. This approach can eliminate the change in the morphology of the prefabricated 3D bioprinted constructs during in vitro construction process, transport during surgery or manipulation of the bioprinted scaffolds to conform to the defect shape [117]. Therefore, in situ bioprinting offers immediate printing of the bioink to the defect site in an anatomically accurate and personalized reconstruction for successful restoration of the tissues [118]. Moreover, it provides an interesting perspective for clinical practice considering that it could eliminate need for the in vitro fabrication phase, which may delay the implantation procedure. In this review, all in situ bioprinting was carried out on calvarial defects using the laser-assisted bioprinting technique. LAB was used to print bioinks containing SCAPs for bone regeneration application. Even though LAB produces high printing resolution and high throughput, this approach is currently not able to fabricate large-scale tissue constructs because of the relatively slow printing speed [18]. However, this technique could be suitable for in situ bioprinting for small defects and relatively flat bones [119].

Therefore, to summarize the current perspectives of advanced research in 3D bioprinting for dental application based on the included studies, some limitations need to be addressed. However, we must acknowledge this is a novel approach and very much in the early stage of development. Firstly, various novel bioinks report promising outcomes on the advancement of customized specific constructs. Nonetheless, there is a wide heterogeneity in bioink composition (type of biomaterials and cells), printing parameters and application in dental tissue engineering which presents a challenge in deciding which bioink is compatible with the best standard of care and restoring the physiological function of the teeth. Secondly, the current research is mostly in vitro studies, hence, they are still in preliminary steps and not yet possible to prove its effectiveness in vivo. In addition, the results from in vivo studies need to be interpreted with great caution considering that the surgically created defects are small. Therefore, fabrication of large 3D printed tissue constructs and implanted into large animal models such as dogs or monkeys would be an optimal study design to better investigate the outcomes of the clinically relevant size and architecture of regenerated tissues. Finally, the ideal research models developed should be able to simulate the dentoalveolar environment since the defect created on the calvarium might not give a true reflection of more complex conditions in the oral cavity. The future prospects of 3D bioprinting are highly promising, and the progress toward the potential development of 3D printed tissues for an individual patient using the patient’s cells needs to be considered for clinical translation. Nevertheless, the implantation of 3D bioprinted tissues in humans, which include living cells and biomaterials, will face regulatory challenges given that the long-term effects such as safety and efficacy in humans are still unknown. Therefore, the ethical, technical and legal issues need to be addressed and regulated by national guidelines to protect the health and well-being of patients before adopting the 3D bioprinting technology into human clinical applications.

## 5. Conclusions

Three-dimensional bioprinted novel bioinks based on natural and synthetic polymers, dECM, cell aggregates and spheroids have shown promising results in dental applications, particularly for periodontal ligament, dentin, dental pulp and bone regeneration. The increasing use of stem cells derived from dental origin can offer a good cell source in oral tissue engineering. In addition, 3D bioprinting brings significant potential in translating advanced tissue engineering into the clinical application by creating regenerative scaffolds tailored to patient-specific requirements. It is hoped that continuous research and advancement in 3D bioprinting, particularly in the techniques and materials used in dental applications, would reach a level of refinement and standard that can be fully integrated into the management and practice in addressing oral healthcare problems.

## Figures and Tables

**Table 1:** Summary of the included studies based on cell-laden bioinks.

Author	Cell-Laden Bioink	Other Biomaterial/ Growth Factor	Cell Types	Bioprinting Strategy	Study Design	Application
Lee et al., 2021 [53]	Collagen	FGF-2	hPDLSCs	Extrusion	In vitro and in vivo	PDL regeneration
Wang et al., 2021 [66]	Collagen	SrCS	Human gingiva fibroblasts	Extrusion	In vitro and in vivo	Periodontal regeneration
Kérourédan et al., 2018 [57]	Collagen type 1	-	SCAPs	LAB	In vitro and in vivo	Bone regeneration
Kérourédan et al., 2019 [58]	Collagen type 1	VEGF	SCAPs and HUVECs	LAB	In vivo	Bone regeneration
Duarte Campos et al., 2020 [60]	Collagen type 1 + agarose	-	DPSCs and HUVECs	Inkjet	In vitro and ex vivo	Dental pulp regeneration
Keriquel et al., 2017 [56]	Collagen type 1 + nHAp	-	Mouse bone marrow stromal precursor D1 cell line	LAB	In vitro and in vivo	Bone regeneration
Moncal et al., 2021 [49]	Collagen + chitosan + β-glycerophosphate + nHAp	rhBMP-2	Rat BMSCs	Extrusion	In vitro	Bone regeneration
Moncal et al., 2022 [50]	Collagen + chitosan + β-glycerophosphate + nHAp	PDGF and BMP-2	Rat BMSCs	Extrusion	In vitro	Bone regeneration
Touya et al., 2022 [59]	Collagen type 1 + TCP (BioRoot RCS^®^, Septodont, Saint-Maur-des- Fossés, France)	-	SCAPs	LAB	In vitro and in vivo	Bone regeneration
Kim et al., 2022 [55]	Collagen type 1 or dECMs + β-TCP	-	DPSCs	Extrusion	In vitro and in vivo	Dental tissue regeneration
Kang et al., 2016 [41]	Gelatin + fibrinogen + HA + glycerol	PCL/TCP	hAFSCs	Extrusion	In vitro and in vivo	Alveolar bone/bone regeneration
Han et al., 2019 [51]	Gelatin + fibrinogen + HA + glycerol	-	DPSCs	Extrusion	In vitro	Dentin/dental pulp regeneration
Han et al., 2021 [52]	Demineralized dentin matrix particles + fibrinogen + gelatin	-	DPSCs	Extrusion	In vitro	Dental tissue regeneration
Kort-Mascort et al., 2021 [68]	Alginate + gelatin + dECMs	-	Human SCC (Cell lines: UM-SCC-12 and UM-SCC-38)	Extrusion	In vitro	Head and neck cancer in vitro model
Tian et al., 2021 [65]	Sodium alginate + gelatin + nHAp	-	hPDLSCs	Extrusion	In vitro	Bone regeneration
Park et al., 2020 [47]	Gelatin + GelMA + HA + glycerol	BMP-mimetic peptide	DPSCs	Extrusion	In vitro	Dental tissue regeneration
Amler et al., 2021 [62]	GelMA	-	Bone-derived MPC/Bone marrow MPC/Periosteal MPC	Stereolithography	In vitro	Bone regeneration
Raveendran et al., 2019 [69]	GelMA	-	hPDLSCs	Extrusion	In vitro	Periodontal regeneration
Kuss et al., 2017 [42]	MeHA + GelMA + HA	PCL/HAp	Porcine stromal vascular fraction from adipose tissue	Extrusion	In vitro	Alveolar bone/bone regeneration
Ma et al., 2015 [63]	GelMA + PEGDA	-	hPDLSCs	Inkjet	In vitro	Periodontal regeneration
Ma et al., 2017 [64]	GelMA + PEGDA	-	Rat PDLSCs	Inkjet	In vitro and in vivo	Alveolar bone regeneration
Amler et al., 2021 [61]	GelMA + PEGDA3400	-	JHOBs and HUVECs	Stereolithography	In vitro	Alveolar bone in vitro model
Lin et al., 2021 [67]	Calsium silicate + GelMA	-	DPSCs	Extrusion	In vitro	Dentin regeneration
Chimene et al., 2020 [46]	GelMA + kCA + nSi (NICE bioink)	-	Human primary bone marrow-derived MSCs	Extrusion	In vitro	Alveolar bone regeneration
Athirasala et al., 2018 [43]	Alginate + dentin matrix	-	SCAPs	Extrusion	In vitro	Dentin/dental pulp regeneration
Walladbegi et al., 2020 [70]	Nanofibrillated cellulose + alginate (CELLINK AB, Gothenburg, Sweden)	β-TCP	hADSCs	Extrusion	In vitro	Bone regeneration
Dubey et al., 2020 [48]	ECM + AMP	-	DPSCs	Extrusion	In vitro	Bone regeneration
Dutta et al., 2021 [54]	Poloxamer-407	-	SCAPs	Extrusion	In vitro	Dental tissue regeneration
Aguilar et al., 2019 [44]	-	-	Mice bone marrow stromal cells	Scaffold-free (Kenzan method)	In vitro	Bone regeneration
Aguilar et al., 2019 [45]	-	-	Mice bone marrow stromal cells	Scaffold-free (Kenzan method)	In vitro	Bone regeneration
Ono et al., 2021 [71]	-	-	Human PDL cell line 1-17	Scaffold-free (Needle array)	In vitro	PDL regeneration

LAB, laser-assisted bioprinting; GelMA, gelatin methacryloyl; PEGDA, poly(ethylene glycol) dimethacrylate; HA, hyaluronic acid; PCL, poly (ε-caprolactone); TCP, tricalcium phosphate; MeHA, methacrylated hyaluronic acid; kCA, kappa-carrageenan; HAp, hydroxyapatite; nHAp, nano-hydroxyapatite; AMP, amorphous magnesium phosphates; nSi, nanosilicates; Poloxamer-407, synthetic copolymer of poly(ethylene glycol) and poly(propylene glycol); ECM, extracellular matrix; dECM, decellularized extracellular matrix; SrCS, strontium-doped calcium silicate; hPDLSCs, human periodontal ligament stem cells; hAFSCs, human amniotic fluid-derived stem cells; SCAPs, human stem cells from apical papilla; DPSCs, human dental pulp stem cells; HUVECs, human umbilical vein endothelial cells; MSCs, mesenchymal stem cells; BMSCs, bone marrow mesenchymal stem cells; hADSCs, human adipose tissue-derived mesenchymal stem cells; JHOBs, jawbone-derived human osteoblasts; MPC, human mesenchymal progenitor cells; SCC, squamous cell carcinoma; VEGF, vascular endothelial growth factor; BMP, bone morphogenetic protein; rhBMP, recombinant bone morphogenetic protein; FGF, fibroblast growth factor; PDGF, platelet-derived growth factor

**Table 2:** Characteristics of the 3D bioprinting techniques.

Author	Cell-Laden Bioink	Type of Polymer	3D Bioprinter	3D Bioprinting Technique	Nozzle Size	Printing Speed	Printing Pressure	Crosslinking Method	Study Outcomes
Lee et al., 2021 [53]	Collagen	Natural	3DX Printer, T and R Biofab Co., Ltd., Siheung, Korea	Extrusion	400 μm ~22G	-	-	Thermal	Connective tissues interface between 3D-printed implants and calvaria bone has periodontal ligament characteristics; however, FGF-2 did not play a role in periodontal regeneration
Wang et al., 2021 [66]	Collagen	Natural	BioScaffolder 3.1, GeSiM, Großerkmannsdorf, Germany	Extrusion	400 μm ~22G	1.5–2 mm/s	10–20 kPa	Physical	Novel bilayer 3D printed SrCS with collagen bioink upregulate angiogenic- and osteogenic-related proteins and factors, and enhanced bone regeneration in vivo
Kérourédan et al., 2018 [57]	Collagen type 1	Natural	LAB workstation (U1026, Inserm, Bordeaux, France)	LAB	-	-	-	-	Potential use of magnetic resonance imaging and bioprinted micron superparamagnetic iron oxide-labeled cells to track cell patterns in vitro and calvarium defect model in mouse
Kérourédan et al., 2019 [58]	Collagen type 1	Natural	LAB workstation (U1026, Inserm, Bordeaux, France)	LAB	-	-	-	-	In situ printing of HUVECs enhance vascularization and bone regeneration in calvarial defects
Duarte Campos et al., 2020 [60]	Collagen type 1 + agarose	Natural	Hand-held bioprinter (DropGun, BlackDrop Biodrucker GmbH, Aachen, Germany)	Inkjet	300 μm ~23G	-	25–250 kPa	Thermal	Handheld in situ bioprinting of cell-loaded collagen-based bioinks demonstrated successful vasculogenesis
Keriquel et al., 2017 [56]	Collagen type 1 + nHAp	Natural	LAB workstation (U1026, Inserm, Bordeaux, France)	LAB	-	250 μm/s	-	-	3D printed disk form of nHAp-collagen and D1 cells (bone marrow stromal precursor cells) showed the formation of mature bone in a calvarial defect model
Moncal et al., 2021 [49]	Collagen + chitosan + β-glycerophosphate + nHAp	Natural	In-house developed MultiArm Bioprinter, Iowa City, IA, USA	Extrusion	22G~410 μm	400 mm/min	80–140 kPa	Thermal and physical	Hybrid intra-operative bioprinting induced bone regeneration with nearly 80% regenerated critical size calvarial bone defect
Moncal et al., 2022 [50]	Collagen + chitosan + β-glycerophosphate + nHAp	Natural	In-house developed MultiArm Bioprinter, Iowa City, IA, USA	Extrusion	22G~410 μm	400 mm/min	80–140 kPa	Thermal and physical	Bioprinted bone constructs with the controlled co-delivery release of growth factors resulted in bone regeneration in critical-sized calvarial defects
Touya et al., 2022 [59]	Collagen type 1 + TCP (BioRoot RCS^®^, Septodont, France)	Natural	LAB workstation (U1026, Inserm, Bordeaux, France)	LAB	-	-	-	-	TCP-based ink demonstrated positive significance upon cell motility, and early osteogenic differentiation in vitro. However, the bioink was not successful in regenerating critical size cranial bone defects in vivo
Kim et al., 2022 [55]	Collagen type 1 or dECMs + β-TCP	Natural	DTR3–2210 T-SG; DASA Robot, Bucheon, Korea	Extrusion	250 μm ~25G	10 mm/s	17–22 kPa	Genipin	The hDPSC-laden bone-derived dECM biocomposite enhanced both osteogenic and odontogenic differentiation in vitro and in vivo
Kang et al., 2016 [41]	Gelatin + fibrinogen + HA + glycerol	Natural	Integrated tissue–organ printing system	Extrusion	300 μm ~23 G	-	50–80 kPa	Thrombin	3D tissue construct provides a favorable microenvironment for osteogenic differentiation of hAFSCs in vitro and showed the formation of mature, vascularized bone tissues in the calvarial bone defect model
Han et al., 2019 [51]	Gelatin + fibrinogen + HA + glycerol	Natural	Integrated tissue–organ printing system	Extrusion	250 μm ~25G	50–90 mm/min	-	Thrombin	Fibrin-based cell-laden bioink demonstrated spatial regulation of DPSC differentiation for the construction of 3D dentin–pulp complexes
Han et al., 2021 [52]	Demineralized dentin matrix particles + fibrinogen + gelatin	Natural	Homemade 3D bioprinter, Ulsan, Korea	Extrusion	300 μm ~23G	50 mm/min	200 kPa	Thrombin	DDMp bioink can be used to fabricate 3D cellular dental constructs and showed significantly improvement in odontogenic differentiation of DPSCs
Kort-Mascort et al., 2021 [68]	Alginate + gelatin + dECMs	Natural	BioScaffolder 3.1, GeSiM, Großerkmannsdorf, Germany	Extrusion	22G ~400 μm	10 ± 2 mm/s	45 ± 10 kPa	Calcium chloride	Cell-laden dECM-based bioink demonstrated tumor spheroids development by squamous cell carcinoma cells with high cell viability and proliferation
Tian et al., 2021 [65]	Sodium alginate + gelatin + nHAp	Natural	3D Bioplotter (EnvisionTEC GmbH, Gladbeck, Germany)	Extrusion	400 μm ~22G	6 mm/s	200 kPa	Calcium chloride	The hPDLSCs-laden bioink demonstrated good biocompatibility, stimulation of cell survival, proliferation and osteoblast
Park et al., 2020 [47]	Gelatin + GelMA + HA + glycerol	Natural	Integrated tissue–organ printing system	Extrusion	330 μm ~23G	150 mm/min	130–160 kPa	Photopolymerization	Novel BMP-GelMA bioink showed high viability, proliferation and odontogenic differentiation of hDPSC
Amler et al., 2021 [62]	GelMA	Natural	Cellbricks GmbH, Berlin, Germany	Stereolithography	-	-	-	Photopolymerization	Periosteum-derived cells showed higher mineralization of print matrix and superior osteogenic potential for 3D bone constructs
Raveendran et al., 2019 [69]	GelMA	Natural	BioScaffolder 3.1, GeSiM, Großerkmannsdorf, Germany	Extrusion	~220 μm 25G	10–12 mm/s	135 kPa	Photopolymerization	The best 3D bioprinting outcome of the periodontal ligament was obtained using 12.5% GelMA concentration with 0.05% LAP extruded through a 25G needle at 135kPa and crosslinking with UV-irradiation
Kuss et al., 2017 [42]	MeHA + GelMA + HA	Natural	3D Bioplotter (EnvisionTEC GmbH, Gladbeck, Germany)	Extrusion	~400 μm 22G	1.8–2.2 mm/s	-	Photopolymerization	Short-term hypoxia (up to 7 days) promoted microvessel formation of SVFC-laden constructs without significantly affecting the cell viability compared to long-term hypoxia (more than 14 days)
Ma et al., 2015 [63]	GelMA + PEGDA	Natural and synthetic	Customer-designed pressure-assisted valve-based bioprinting system	Inkjet	150 μm ~30G	-	40–60 kPa	Photopolymerization	Volume ratios of GelMA to PEG bioink have an impact on cell viability and spreading of hPDLSCs. The increasing ratio of PEG leads to a decrease in hPDLSCs viability and spreading area
Ma et al., 2017 [64]	GelMA + PEGDA	Natural and synthetic	Customer-designed pressure-assisted valve-based bioprinting system	Inkjet	150 μm ~30G	-	50 kPa	Photopolymerization	An increase in the volume ratio of 3D GelMA-PEGDA in vitro resulted in an increase in cell proliferation, spreading and osteogenic differentiation of PDLSCs. New bone formation was observed in the alveolar defect treated with 3D bioprinted PDLSC hydrogel in a rat model
Amler et al., 2021 [61]	GelMA + PEGDA3400	Natural and synthetic	Cellbricks GmbH, Berlin, Germany	Stereolithography	-	-	-	Photopolymerization	3D bioprinted constructs containing primary JHOBs with vasculature-like channel structures comprising endothelial cells demonstrated the survival of both cells and mineralization of the bone matrix
Lin et al., 2021 [67]	Calsium silicate + GelMA	Natural	BioX, CELLINK, Gothenburg, Sweden	Extrusion	30G~150 μm	20 mm/s	180 kPa	Photopolymerization	Calcium silicate/GelMA scaffolds enhanced mechanical properties and odontogenesis of hDPSCs
Chimene et al., 2020 [46]	GelMA + kCA + nSi (NICE bioink)	Natural	Modified ANET A8 3D printer, Shenzhen, China	Extrusion	400 μm ~22G	15 mm/s	-	Photopolymerization	3D NICE cell-laden bioink demonstrated the ability to form osteo-related mineralized ECM without the growth factor
Athirasala et al., 2018 [43]	Alginate + dentin matrix	Natural	Hyrel 3D, Norcross, GA, USA	Extrusion	Coaxial: 26–19G	-	-	Calcium chloride	Cell-laden alginate and dentin matrix enhances odontogenic differentiation of SCAPs
Walladbegi et al., 2020 [70]	Nanofibrillated cellulose + alginate (CELLINK AB, Gothenburg, Sweden)	Natural	Inkredible, CELLINK AB, Gothenburg, Sweden	Extrusion	Coaxial: 22–16G	-	75 kPa and 85 kPa	Calcium chloride	A coaxial needle enables the printing of a stable scaffold with viable hADSCs
Dubey et al., 2020 [48]	ECM + AMP	Natural	3DDiscovery, regenHU, Villaz-St-Pierre, Switzerland	Extrusion	-	15–20 mm/s	30–50 kPa	Physical	ECM/AMP-bioprinted constructs demonstrated osteogenic differentiation of DPSCs without the need for chemical inducers
Dutta et al., 2021 [54]	Poloxamer-407	Synthetic	CELLINK BIO-X 3D printer, Gothenburg, Sweden	Extrusion	27G	5 mm/s	35 kPa	Photopolymerization	3D bioprinted poloxamer hydrogels with low voltage–frequency electromagnetic fields stimulation (5V-1 Hz, 0.62 mT) enhance the SCAPs viability and osteogenic potential
Aguilar et al., 2019 [44]	-	-	Regenova Bio 3D Printer, Cyfuse K.K, Tokyo, Japan	Scaffold-free (Kenzan method)	-	-	-	-	Centrifugation cell method generated tighter BMSC spheroid formation with the optimal technique of 40k cells aggregate under 150-300G
Aguilar et al., 2019 [45]	-	-	Regenova Bio 3D Printer, Cyfuse K.K, Tokyo, Japan	Scaffold-free (Kenzan method)	-	-	-	-	Optimization of scaffold-free bioprinting resulted in a reduction in print times, the use of bioprinting nozzles and fabrication of more robust constructs
Ono et al., 2021 [71]	-	-	Regenova Bio 3D Printer, Cyfuse K.K, Tokyo, Japan	Scaffold-free (Needle array)	240 μm ~26G	-	-	-	3D bioprinted tubular structures and hydroxyapatite core materials exhibited high cell viability, collagen fibers and strongly expressed factors associated with periodontal ligament tissues

3D, three-dimensional; LAB, laser-assisted bioprinting; USA, United States of America; GelMA, gelatin methacryloyl; PEG, poly(ethylene glycol); PEGDA, poly(ethylene glycol) dimethacrylate; HA, hyaluronic acid; TCP, tricalcium phosphate; MeHA, methacrylated hyaluronic acid; kCA, kappa-carrageenan; nHAp, nano-hydroxyapatite; AMP, amorphous magnesium phosphates; nSi, nanosilicates; Poloxamer-407, synthetic copolymer of poly(ethylene glycol) and poly(propylene glycol); ECM, extracellular matrix; dECM, decellularized extracellular matrix; LAP, lithium phenyl-2,4,6-trimethylbenzoylphosphinate; DDMp, demineralized dentin matrix particles; SrCS, strontium-doped calcium silicate; SVFC, stromal vascular fraction derived cells; hPDLSCs, human periodontal ligament stem cells; hAFSCs, human amniotic fluid-derived stem cells; SCAPs, human stem cells from apical papilla; DPSCs, human dental pulp stem cells; HUVECs, human umbilical vein endothelial cells; MSCs, mesenchymal stem cells; BMSCs, bone marrow mesenchymal stem cells; hADSCs, human adipose tissue-derived mesenchymal stem cells; JHOBs, jawbone-derived human osteoblasts; FGF, fibroblast growth factor; UV, ultraviolet.

**Table 3:** Characteristics of cell types in 3D bioprinting application.

Author	Cell Type	Cell Densities	Max Cell Viability (%)	3D Bioprinting Technique	Targeted Tissue
Han et al., 2019 [51]	DPSCs	3 × 10^6^ cells/mL	>90	Extrusion	Dentin/dental pulp
Park et al., 2020 [47]	DPSCs	-	>90	Extrusion	Dental tissue
Dubey et al., 2020 [48]	DPSCs	1 × 10^6^ cells/mL	>90	Extrusion	Bone
Han et al., 2021 [52]	DPSCs	3 × 10^6^ cells/mL	>95	Extrusion	Dental tissue
Lin et al., 2021 [67]	DPSCs	5 × 10^6^ cells/mL	-	Extrusion	Dentin/pulp
Kim et al., 2022 [55]	DPSCs	1 × 10^7^ cells/mL	>95	Extrusion	Dental tissue
Duarte Campos et al., 2020 [60]	DPSCs and HUVECs	3 × 10^6^ cells/mL (both type of cells)	-	Inkjet	Dental pulp
Ma et al. 2015 [63]	hPDLSCs	1 × 10^6^ cells/mL	82.4 ± 4.7	Inkjet	Periodontal ligament
Raveendran et al., 2019 [69]	hPDLSCs	2.0 × 10^6^ cells/mL	>70	Extrusion	Periodontal ligament
Lee et al., 2021 [53]	hPDLSCs	1 × 10^7^ cells/mL	-	Extrusion	Periodontal ligament
Tian et al., 2021 [65]	hPDLSCs	-	-	Extrusion	Bone
Ma et al., 2017 [64]	Rat PDLSCs	1 × 10^6^ cells/mL	~90	Inkjet	Bone
Athirasala et al., 2018 [43]	SCAPs	0.8 × 10^6^ cells/mL	>90%	Extrusion	Dentin/dental pulp
Kérourédan et al., 2018 [57]	SCAPs	7 × 10^7^ cells/mL		LAB	Bone
Dutta et al., 2021 [54]	SCAPs	2.5 × 10^4^ cells/mL	-	Extrusion	Dental tissue
Touya et al., 2022 [59]	SCAPs	2 × 10^3^ cells/mL	-	LAB	Bone
Kérourédan et al., 2019 [58]	SCAPs and HUVECs	7 × 10^7^ cells/mL	-	LAB	Bone
Wang et al., 2021 [66]	Human gingiva fibroblasts	5 × 10^5^ cells/mL	-	Extrusion	Periodontal ligament/Bone
Ono et al., 2021 [71]	Human PDL cell line 1–17	2.5 × 10^4^ cells/mL	-	Scaffold-free (Kenzan method)	Periodontal ligament
Kort-Mascort et al., 2021 [68]	Human SCC (Cell lines: UM-SCC-12 and UM-SCC-38)	1 × 10^6^ cells/mL	>95	Extrusion	Dental tissue
Chimene et al., 2020 [46]	Human primary bone marrow-derived MSCs	-	-	Extrusion	Bone
Amler et al., 2021 [62]	Bone-derived MPC/Bone marrow MPC/Periosteal MPC	20 × 10^6^ cells/mL	-	Stereolithography	Bone
Moncal et al., 2021 [49]	Rat BMSCs	5 × 10^6^ cells/mL	>95	Extrusion	Bone
Moncal et al., 2022 [50]	Rat BMSCs	8 × 10^5^ cells/mL	>95	Extrusion	Bone
Aguilar et al., 2019 [44]	Mice bone marrow stromal cells	-	-	Scaffold-free (Kenzan method)	Bone
Aguilar et al., 2019 [45]	Mice bone marrow stromal cells	-	-	Scaffold-free (Kenzan method)	Bone
Keriquel et al., 2017 [56]	Mouse bone marrow stromal precursor D1 cell line	120 × 10^6^ cells/mL	-	LAB	Bone
Amler et al., 2021 [61]	JHOBs and HUVECs	20 × 10^6^ cells/mL	-	Stereolithography	Bone
Walladbegi et al., 2020 [70]	hADSCs	4 × 10^6^ cells/mL	~80	Extrusion	Bone
Kuss et al., 2017 [42]	Porcine stromal vascular fraction from adipose tissue	4 × 10^6^ cells/mL	-	Extrusion	Bone
Kang et al., 2016 [41]	hAFSCs	5 × 10^6^ cells/mL	91 ± 2	Extrusion	Bone

LAB, laser-assisted bioprinting; hPDLSCs, human periodontal ligament stem cells; hAFSCs, human amniotic fluid-derived stem cells; SCAPs, human stem cells from apical papilla; DPSCs, human dental pulp stem cells; HUVECs, human umbilical vein endothelial cells; MSCs, mesenchymal stem cells; BMSCs, bone marrow mesenchymal stem cells; hADSCs, human adipose tissue-derived mesenchymal stem cells; JHOBs, jawbone-derived human osteoblasts; MPC, human mesenchymal progenitor cells; SCC, squamous cell carcinoma.

**Table 4:** Summary of animal model characteristics.

Author	Animal Model	Sex	Age	Weight	Defect Area	Defect Size	In Situ Printing	Time of Sacrifice
Keriquel et al., 2017 [56]	Balb/c mice	Female	12 weeks	19–20 g	Calvarium	3.3 mm diameter	Yes	8 weeks
Kérourédan et al., 2018 [57]	NOG mice	Female	10 weeks	25–26 g	Calvarium	3.3 mm diameter	Yes	-
Kérourédan et al., 2019 [58]	NSG mice	Female	10 weeks	25–26 g	Calvarium	3.3 mm diameter	Yes	4 or 8 weeks
Touya et al., 2022 [59]	NSG mice	Female	8 weeks	-	Calvarium	3.3 mm diameter	Yes	4 weeks or 8 weeks
Kang et al., 2016 [41]	Sprague Dawley rats	-	-	250–300 g	Calvarium	8 mm diameter, 1.2 mm depth	No	20 weeks
Lee et al., 2021 [53]	Athymic rats	Male	9 weeks	-	Calvarium	8 mm diameter, 1.5 mm depth	No	6 weeks
Wang et al., 2021 [66]	New Zealand white rabbit	Female	-	2 kg	Calvarium	7 mm diameter, 8 mm depth	No	12 weeks
Ma et al., 2017 [64]	Sprague Dawley rats	-	33 months	230–250 g	Alveolar bone	4 mm length × 3 mm width × 2 mm height	No	3 and 6 weeks
Kim et al., 2022 [55]	Athymic nude mice	-	-	-	Dorsal subcutaneous	-	No	8 weeks
